# Macular Bruch’s membrane defect and dome-shaped macula in high myopia

**DOI:** 10.1371/journal.pone.0178998

**Published:** 2017-06-01

**Authors:** Yuxin Fang, Jost B. Jonas, Tae Yokoi, Kejia Cao, Kosei Shinohara, Kyoko Ohno-Matsui

**Affiliations:** 1 Department of Ophthalmology and Visual Science, Tokyo Medical and Dental University, Tokyo, Japan; 2 Department of Ophthalmology, Medical Faculty Mannheim of the Ruprecht-Karls-University of Heidelberg, Heidelberg, Germany; University of Manchester, UNITED KINGDOM

## Abstract

**Purpose:**

To examine an association between macular Bruch’s membrane defects (MBMD) and a dome-shaped appearance of the macula (DSM).

**Design:**

Retrospective, observational case series study.

**Methods:**

The study included highly myopic individuals who were consecutively examined between May 2014 and December 2015. The patients underwent swept-source optical coherence tomography (OCT) for visualization of DSM and MBMDs defined as Bruch´s membrane defects located at a distance of maximal 1500 μm from the foveola.

**Results:**

Out of 1983 highly myopic eyes (1057 patients), 166 eyes (8.4%; 95% confidence interval (CI):7.2%,9.6%)) showed a DSM and 534 eyes showed a MBMD. In multivariate binary regression analysis, higher prevalence of DSM was associated with a higher prevalence of a MBMD (P<0.001; OR: 1.96; 95%CI: 1.40, 2.75) after adjusting for longer axial length (P<0.001; odds ratio (OR): 1.27; 95%CI: 1.16, 1.38). In eyes with a DSM partially surrounded by a MBMD, the retina, retinal pigment epithelium (RPE) and choroid appeared relatively unchanged in the central region with Bruch´s membrane (BM) preserved. In the ring-like BM-free region surrounding the central prominent island of the DSM, the RPE, the outer and middle retinal layers, the choriocapillaris and the middle-sized choroidal vessel layer were absent. In association with a DSM, three MBMD types were differentiated: MBMDs in patchy chorioretinal atrophy, MBMDs in choroidal neovascularization-related macular atrophy, and MBMDs as temporally extending large parapapillary gamma zone.

**Conclusions:**

Presence of a DSM was significantly associated with the presence of MBMDs. The morphology of the DSM in association with MBMDs may be associated with a focal relaxation of the posterior sclera, no longer pushed outward by an expanding BM but allowed to partially bulge inward, leading to the formation of a DSM.

## Introduction

Dome-shaped macula (DSM) is an inward protrusion of the macula as visualized by optical coherence tomography (OCT).[1, 2] Imamura, Spaide and coworkers reported that a DSM was associated with, and caused by, a local thickening of the subfoveal sclera.[3] It was postulated that the local thickening of the subfoveal sclera was an adaptive or compensatory response to the defocus of the image on the fovea in highly myopic eyes. By the relative inward protrusion of the foveola in eyes with a DSM, the optical axis got less elongated and the image on the fovea was less myopically defocused while the perifoveal regions further expanded backward.[4–6] The mechanism leading to the relative thickening of the subfoveal sclera remained elusive.

Bruch’s membrane (BM) is composed of 5 layers: 1) the basement membrane of the retinal pigment epithelium (RPE), 2) an inner collagenous zone, 3) an elastic layer, 4) an outer collagenous zone, and 5) the basement membrane of the choriocapillaris.[7] It was generally assumed that BM in highly myopic eyes remained intact in the area of a myopic macular chorioretinal atrophy despite the loss of RPE, closure of choriocapillaris and loss of photoreceptors.[8] However, defects of BM in the macular region were recently in a histological study of highly myopic eyes.[9] These macular BM defects (MBMDs) were accompanied by a complete loss of RPE and choriocapillaris and an almost complete loss of the outer and middle retinal layers and of the middle-sized choroidal vascular layer. Ohno-Matsui et al. recently reported that MBMDs occurred in association with two different macular lesions specific to pathologic myopia, i.e. with myopic choroidal neovascularization (CNV)-related macular atrophy and with patchy chorioretinal atrophy.[10, 11] Macular lesions which had previously been considered to be chorioretinal atrophies were not simply atrophy but were a hole of BM. In addition to the axial elongation-associated development of new BM defects in the macular area, the physiological defect in BM in the region of the optic nerve head can enlarge in axially elongated eyes so that the peripapillary region is no longer covered by BM.[12] This BM free region, which can also be visualized by OCT, has been called parapapillary gamma zone.[12]

Since MBMDs may represent a region of low biomechanical strength at the posterior pole and since the etiology of DSM has remained unresolved yet, we addressed the hypothesis that DSM was associated with MBMDs, and conducted this study to assess a potential relationship between both entities in highly myopic eyes.

## Materials and methods

The retrospective, observational case series study included patients with high myopia who had consecutively been examined by swept-source OCT (DRI-OCT; Topcon, Tokyo, Japan) in the High Myopia Clinic of the Tokyo Medical and Dental University between May 2014 and December 2015. High myopia was defined by a myopic refractive error of >-8 diopters or by an axial length of ≥26.5 mm. Eyes with fundus features of pathologic myopia (defined as category 2 of myopic maculopathy as described by the META-PM study [13]) were enrolled. All patients underwent a comprehensive ophthalmological examination including refractometry, measurement of axial length (IOL Master; Carl Zeiss Meditec Co., Jena, Germany), color fundus photography, photography of fundus autofluorescence and OCT. Exclusion criteria included an insufficient quality of the OCT images due to media opacities such as dense cataract, and previous vitreoretinal surgery since the latter might have affected the scleral curvature. Low image quality was defined as insufficient visualization of the RPE line in the macular area, unless the RPE was damaged due to a pathological process. In agreement with the tenets of Declaration of Helsinki, the study was approved by the Ethics Committee of Tokyo Medical and Dental University. All data were fully anonymized before access by the researchers.

The diagnosis of a DSM and of MBMDs was based on the swept-source OCT images. The OCT scanning protocols included a length of the radial scans of 6 mm or 9 mm with 12 equal meridian scans centered on the fovea. The swept-source OCT device applied in the study had an A-scan repetition rate of 100,000 Hz and its light source operated in the wavelength range of 1 μm. All OCT images were examined by two retina specialists (YXF, KOM), in a first step separately, and then jointly. An agreement in the diagnosis of DSM and MBMDs between both examiners was obtained for all images included into the study.

In accordance with previous studies, DSM was defined as an inward bulging of the RPE line with a maximal height >50 μm above a base line connecting the RPE lines on both sides outside of the DSM.[2, 14] A MBMD was defined as a defect in BM the edge of which was located within a distance of <1500 μm from the foveola on at least one of the 12 radial OCT scans. As in earlier studies, we differentiated between three types of MBMDs: MBMDs in CNV-related macular atrophy,[10] MBMDs in patchy chorioretinal atrophy[11] and a MBMDs as a large parapapillary gamma zone extending into the macular region.[9, 12, 15]

For statistical analyses, we applied a statistical software package (SPSS for Windows, version 22.0, IBM-SPSS, Chicago, IL, USA). We first described the distribution of the main parameters by calculating their means and standard deviations. As a second step, we compared the assessed parameters between eyes with DSM and eyes without DSM in a univariate analysis using the Mann-Whitney U test. Frequencies were compared using the Chi-square test. As a third step, we carried out a multivariate binary regression analysis with the presence of DSM as dependent variable and all those parameters as independent variables, which were significantly associated with the presence of DSM in the univariate analysis. We then dropped step by step those parameters which either showed a collinearity or which were no longer significantly associated with the presence of DSM. We calculated the odds ratio (OR) and the 95% confidence intervals (CI). A P-value of <0.05 was considered to be statistically significant.

## Results

In the study period, 2018 highly myopic eyes (1075 patients) were consecutively examined out of which 35 eyes were excluded because of poor quality of OCT images, so that the study eventually included 1983 eyes of 1057 patients with a mean age of 61.5 ± 15.4 years (range: 4–94 years) and a mean axial length of 29.8 ± 1.9 mm (range: 23.5–36.4 mm). The fundus of the eye with the shortest axial length (23.5 mm) in the study population showed a patchy chorioretinal atrophy, so that, despite its relative short axial length, it remained in the study population.

Out of the 1983 highly myopic eyes, 166 eyes (8.4%; 95%CI: 7.2%, 9.6%) of 121 patients showed a DSM, which occurred bilaterally in 45 patients. A typical round dome was found in 4 eyes (2.4%), 152 eyes (91.6%) had a horizontally oriented dome, and 7 eyes (4.2%) showed a vertically oriented dome; in 3 eyes (1.8%) the dome was obliquely oriented. The mean age of the patients with a DSM was 63.0 ± 13.7 years (range: 12–93 years) and the mean axial length of their eyes was 30.7 ± 1.8 mm (range: 26.2–35.9 mm). The eyes with DSM as compared to the eyes without DSM were significantly more myopic (-15.9 ± 3.8 diopters versus -13.2 ± 4.1 diopters; P<0.001), had a significantly longer axial length (30.7 ± 1.8 mm versus 29.7 ± 1.9 mm; P<0.001), and had a worse best corrected visual acuity (expressed in the negative decadic logarithm of the minimal angle of resolution, logMAR) (0.38 ± 0.43 versus 0.30 ± 0.47; P = 0.03), while there was no significant difference in age (63.2 ± 13.7 years versus 61.4 ± 15.6 years; P = 0.33) between both groups.

Out of the 1983 highly myopic eyes, 534 eyes (26.9%; 95%CI: 24.9%, 28.9%) of 402 patients showed a MBMD, which occurred bilaterally in 132 patients. The mean age of the patients with a MBMD was 69.1 ± 11.3 years (range: 11–94 years) and the mean axial length of their eyes was 30.2 ± 2.1 mm (range: 23.5–35.9 mm). The eyes with MBMD as compared to the eyes without MBMD had a significantly longer axial length (30.2 ± 2.1 mm versus 29.6 ± 1.8 mm; P<0.001), had a significantly older age (69.1 ± 11.3 years versus 58.8 ± 15.8 years; P<0.001), and had a worse best corrected visual acuity (0.67 ± 0.54 versus 0.17 ± 0.35; P<0.001).

**Fig 1:**
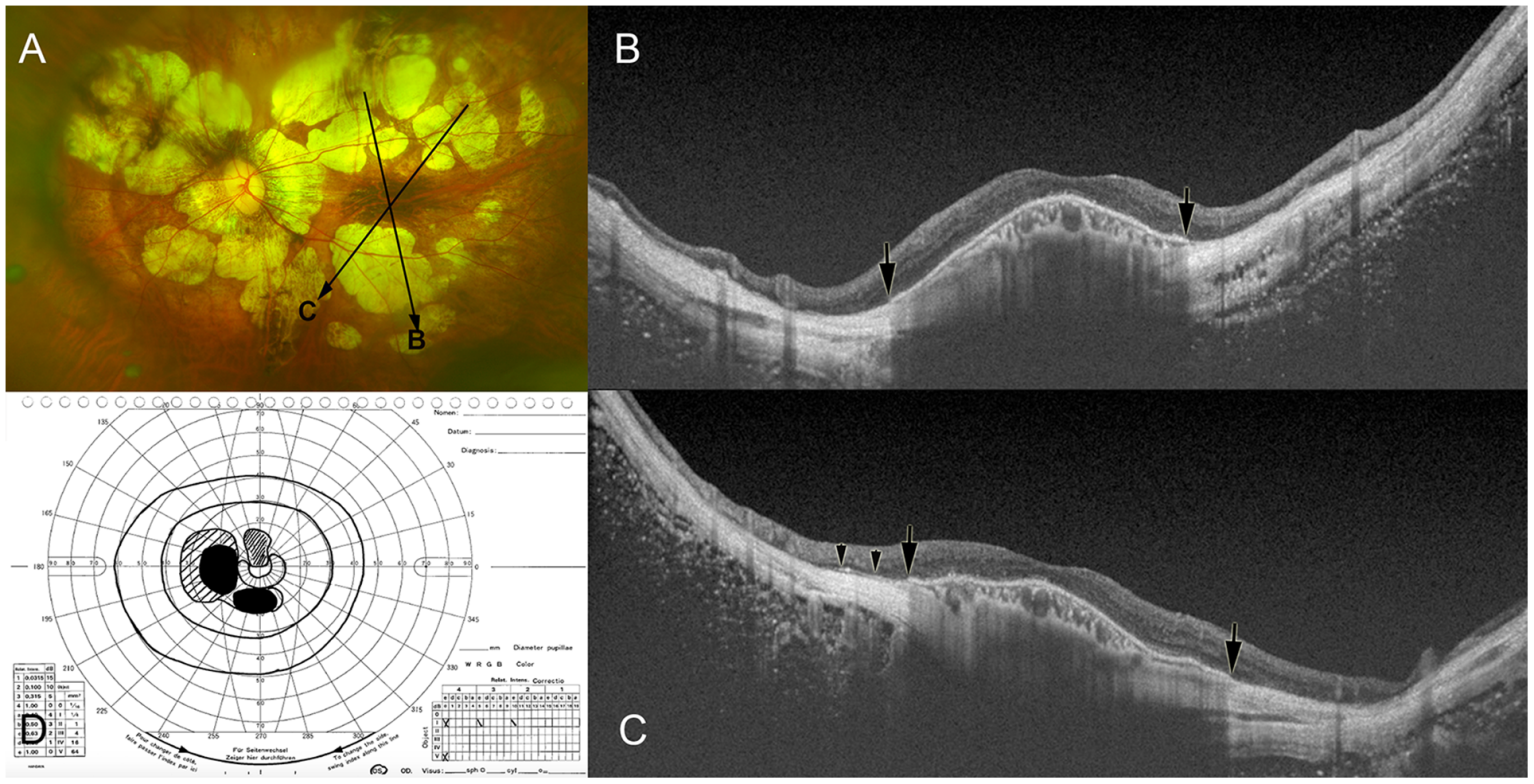
Dome-shaped macula (DSM) co-existing with macular Bruch’s membrane defects (MBMD) associated with patchy atrophy. (A) Left fundus of a 65-year-old woman with an axial length of 33.2 mm shows multiple patches of whitish, well-defined patchy atrophy superior and inferior to the macula. Two long arrows show the scanned lines examined by optical coherence tomography (OCT) in the images (B) and (C), respectively. (B) Oblique OCT section across the fovea shows an inward protrusion due to DSM. Bruch’s membrane remained at the center of macula, but stopped abruptly (arrow) on both sides of the dome. (C) Oblique OCT section across the fovea shows the inward protrusion of the macula due to DSM. Bruch’s membrane remained at the center of macula, but stopped abruptly (arrow) on both sides of the dome. The remnants of Bruch’s membrane are seen near the edge of the MBMD (arrowheads). (D) Print-out of kinetic Goldmann perimetry showing visual field defects almost circular around the fixation region within the central region of 30°; the scotoma in the superior and inferior paracentral region correspond to the location of patchy atrophy.

**Fig 2:**
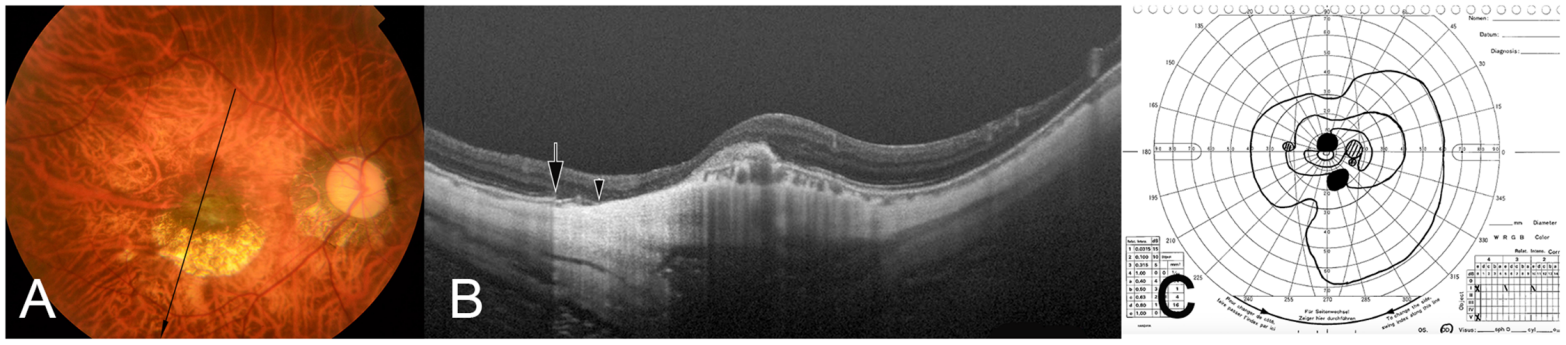
Dome-shaped macula (DSM) co-existing with macular Bruch’s membrane defects(MBMD) associated with myopic choroidal neovascularization (CNV)-related macular atrophy. (A) Right fundus of a 51-year-old woman with an axial length of 31.7mm shows the CNV-related macular atrophy inferior to the pigmented CNV scar. (B) An oblique OCT section across the fovea demonstrates the inward protrusion of the macula due to DSM. CNV is also seen as subretinal elevated lesion. The end of retinal pigment epithelium is indicated by an arrow. The end of Bruch’s membrane is shown by an arrowhead, and BM is absent between this end of BM and the lower edge of the scarred CNV. (C) Print-out of kinetic Goldmann perimetry demonstrating perimetric defects in the superior paracentral region corresponding to the location CNV-related macular atrophy. The nasally located visual field defect may be caused by the co-existing myopic optic neuropathy.

**Fig 3:**
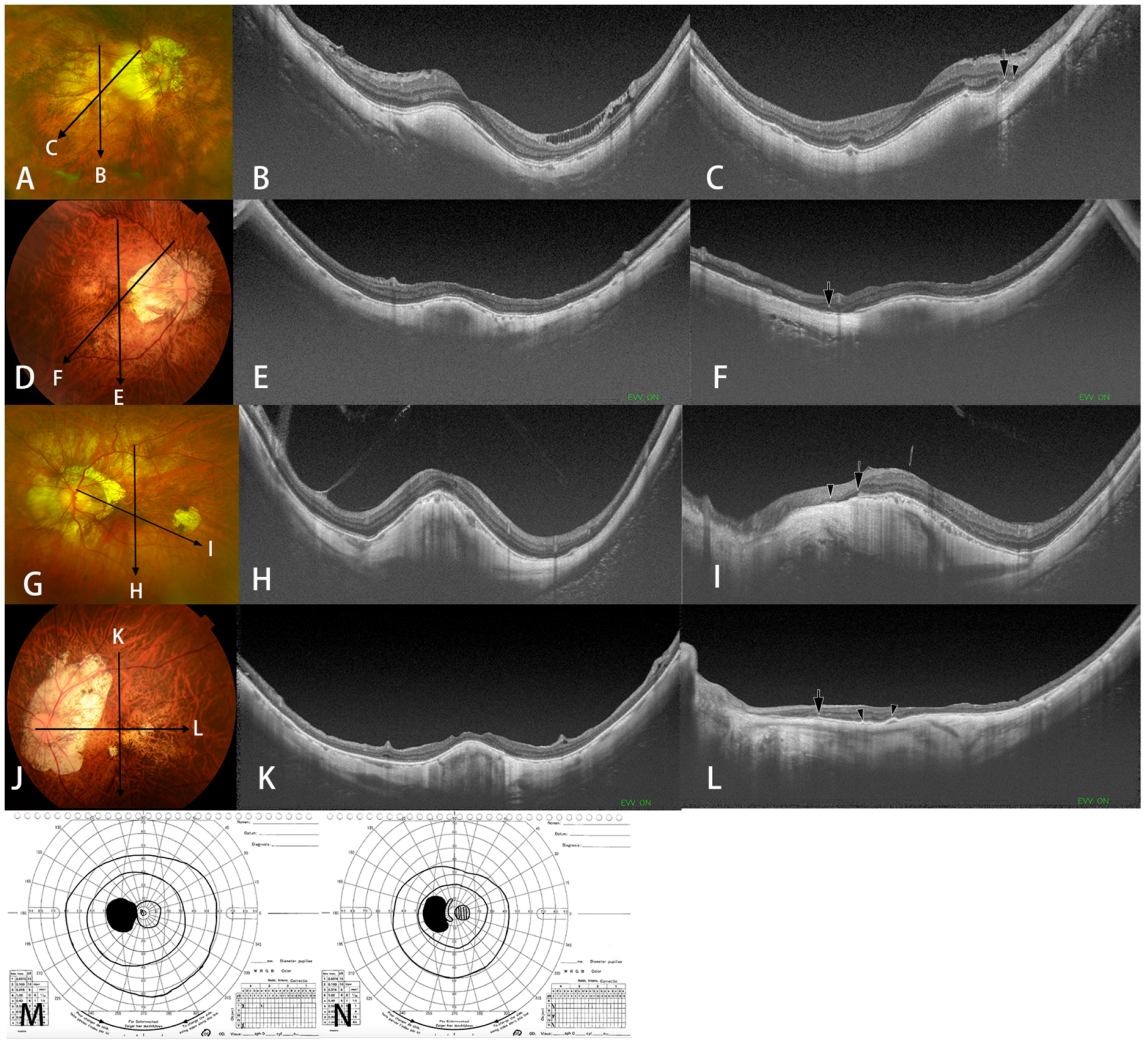
Dome-shaped macula (DSM) co-existing with macular Bruch’s membrane defects (MBMD) associated with large parapapillary atrophy (PPA). (A-C) Right eye of a 73-year-old woman with an axial length of 32.1mm. (A) The right fundus shows a large PPA extending close to the fovea. Myopic maculopathy of category 2 [13] (diffuse atrophy) is present. (B) A vertical OCT section across the fovea shows an inward protrusion of the macula due to DSM. (C) An oblique OCT section across the fovea shows the end of the retinal pigment epithelium (arrow) and the end of Bruch’s membrane (arrowhead) along the temporal margin of the PPA. (D-F) Right eye of a 73-year-old woman with an axial length of 30.1 mm. (D) Right fundus shows a large PPA extending close to the fovea. Myopic maculopathy of category 2 [13] (diffuse atrophy) is present. (E) A vertical OCT section across the fovea shows an inward protrusion of the macula due to DSM. (F) An oblique OCT section across the fovea shows the end of BM (arrow) along the temporal margin of the PPA. (G-I) Left eye of a 55-year-old man with an axial length of 31.3 mm. (G) The left fundus shows a large PPA extending close to the fovea. Patchy atrophy is present temporal to the fovea. (H) A vertical OCT section across the fovea shows an inward protrusion of the macula. (I) Oblique OCT section across the fovea shows the end of the RPE (arrow) and the end of BM (arrowhead) along the temporal margin of the PPA. (J-L) Right eye of a 79-year-old woman with an axial length of 28.0mm. (J) Right fundus shows a large PPA whose edge is close to the fovea. A small area of patchy atrophy is also seen inferior nasal to the fovea. (K) A vertical OCT section across the fovea shows an inward protrusion of macula. (L) In the horizontal OCT section, the end of BM is observed (arrow) and two parts of RPE was curved as shown (arrowhead). (M-N) Print-out of kinetic Goldmann perimetry showing an enlarged blind spot corresponding to the large PPA shown in images 3M and 3N, respectively.

**Fig 4:**
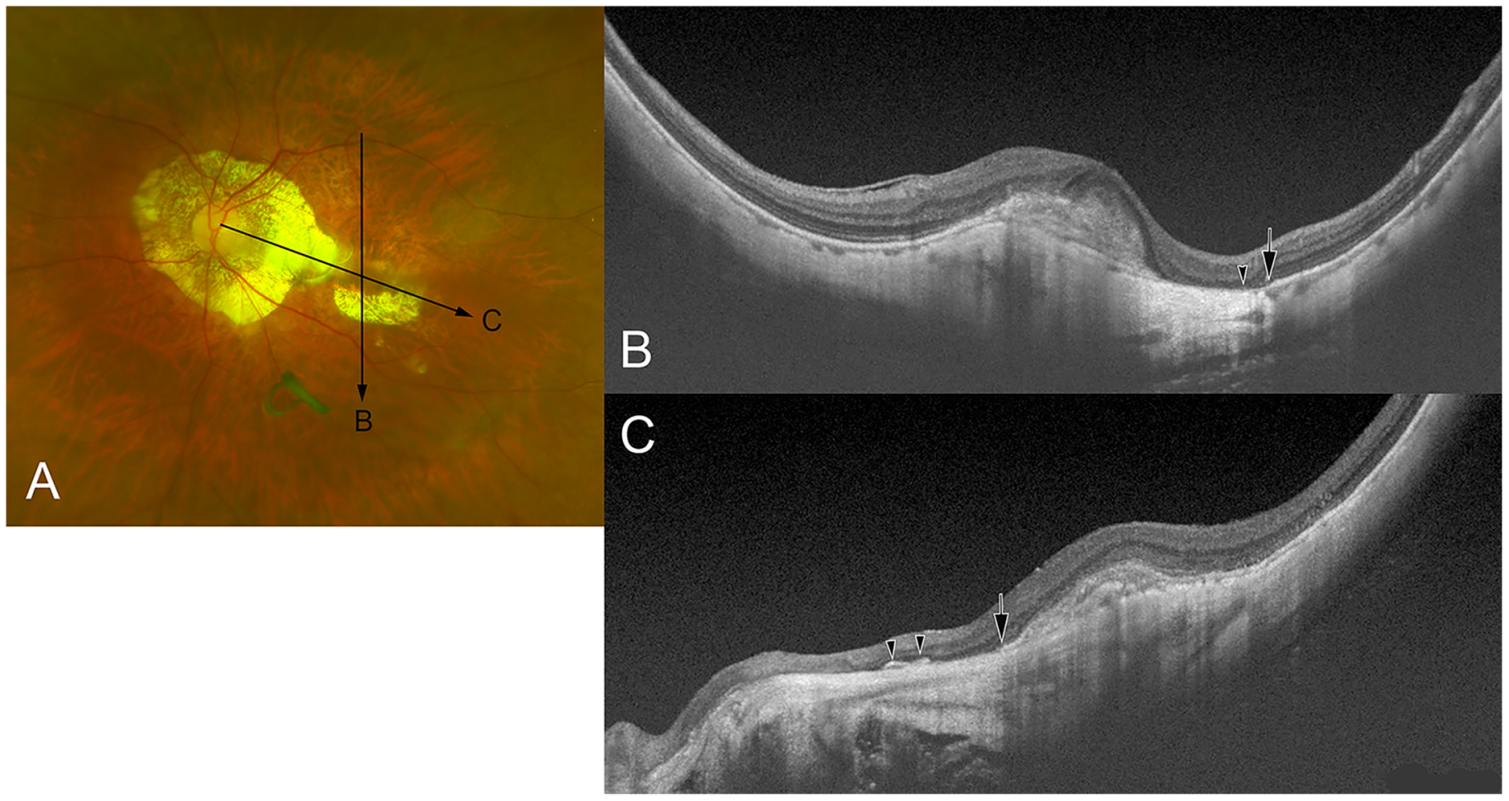
DSM co-existing with macular Bruch’s membrane defects (MBMD) associated with both large parapapillary atrophy (PPA) and myopic choroidal neovascularization (CNV)-related macular atrophy. (A) The left fundus of a 76-year-old woman with an axial length of 28.8 mm shows a CNV-related macular atrophy inferior to the pigmented CNV scar in the fovea. In addition, a large PPA the temporal edge of which is located in proximity to the CNV is detected. (B) A vertical OCT section across the fovea shows the end of the retinal pigment epithelium (RPE; arrow) and the end of Bruch’s membrane (arrowhead). The Bruch’s membrane is absent between the point indicated by an arrowhead and the lower edge of the CNV. (C) An oblique OCT section across the fovea shows the end of the RPE (arrow) along the temporal margin of the PPA. Bruch’s membrane is also absent in the area of the PPA, besides sporadically remaining fragments (arrowheads).

Within the group of eyes with a DSM, one or more MBMDs were found in 68 out of the 166 eyes (41.0%). The prevalence of MBMDs was significantly higher in the group of eyes with a DSM than that in the group of eyes without DSM (68 / 166 (41.0%) versus 466 / 1817 eyes (25.6%)); (P<0.001). Within the group of eyes with a DSM, there were 42 MBMDs associated with patchy atrophy (Fig 1), 19 MBMDs associated with CNV-related macular atrophy (Fig 2), and 12 MBMDs as an extension of a large parapapillary gamma zone (Fig 3). Five eyes had two different patterns of MBMDs in the same eye (Fig 4): One eye with parapapillary gamma zone and patchy atrophy, two eyes with gamma zone and CNV-related macular atrophy, and one eye with patchy atrophy and CNV-related macular atrophy. Among the 12 eyes with MBMDs as an extension of a large parapapillary gamma zone, 9 eyes had MBMDs solely due to gamma zone. All of these 9 eyes had myopic maculopathy of category 2 (i.e., diffuse chorioretinal atrophy).

In univariate analysis, higher frequency of DSMs was associated with longer axial length (P<0.001; OR: 1.30; 95%CI: 1.20, 1.41), presence of a MBMD (P<0.001; OR: 2.11; 95%CI: 1.53, 2.93), lower best corrected visual acuity (logMAR) (P = 0.03; OR: 1.42; 95%CI: 1.04, 1.95), and more myopic refractive error (P<0.001; OR: 0.87; 95%CI: 0.83, 0.91). It was not significantly correlated with age (P = 0.15). The forward stepwise regression analysis included the presence of a DSM as dependent variable and axial length, presence of a MBMD, best-corrected visual acuity, and refractive error as independent variables. Due to its collinearity with axial length, we first dropped refractive error from the list of independent parameters. Due to a lack of statistical significance, we then dropped step by step best corrected visual acuity (P = 0.71) and age (P = 0.55). In the final model, presence of a DSM was significantly associated with longer axial length (P<0.001; OR: 1.27; 95%CI: 1.16, 1.38) and presence of a MBMD (P<0.001; OR: 1.96; 95%CI: 1.40, 2.75). Similar results were obtained in a backward stepwise regression analysis.

Higher prevalence of MBMD was significantly associated (univariate analysis) with higher prevalence of DSM (P<0.001), longer axial length (P<0.001), lower best corrected visual acuity (P<0.001), and higher age (P<0.001). The stepwise binary regression analysis included the presence of a MBMD as dependent variable and as independent variables all those parameters which were significantly associated with the prevalence of MBMDs in the univariate analysis. It revealed that higher prevalence of MBMD was significantly associated with higher presence of a DSM (P<0.001; OR: 1.99; 95%CI: 1.36, 2.90) after adjusting for longer axial length (P<0.001; OR: 1.20; 95%CI: 1.13, 1.28), older age (P<0.001; OR: 1.04; 95%CI: 1.03, 1.05) and lower best corrected visual acuity (logMAR) (P<0.001; OR: 7.76; 95%CI: 5.91, 10.2).

In the eyes with DSM and a MBMD associated with patchy atrophy, BM was present in the center of the macula, but ended on both sides of the dome in at least one of the 12 radial OCT scan sections (Fig 1). It appeared that in these eyes a ring-like MBMD partially encircled the DSM which looked like as an emerging island on the OCT images. In the center of the DSM, the thickness of the choroid and of the retina were within the normal range, the layer of the choriocapillaris (defined as choroid minus large vessel layer and minus medium-sized vessel layer) was present, the RPE did not show interruptions or other irregularities, and the inner segment-outer segment line and the outer limiting membrane line were continuous (Fig 1). Outside of the DSM in the region of the MBMD, RPE and choriocapillaris were not present nor were the medium-sized choroidal vessel layer, the layer of the photoreceptors and the middle retinal layer. The markedly thinned retinal tissue consisted mostly of retinal nerve fiber layer.

In the eyes with a DSM and a MBMD associated with a CNV-related macular atrophy, MBMDs were observed mainly around the CNV, while in the area of the CNV it was difficult to clearly distinguish BM from the scar tissue. In 11 out of the 19 eyes with a MBMD associated with a CNV-related macular atrophy, at least some fragments of BM were detected under the scarred CNV (Fig 2).

In the eyes with a DSM and a MBMD associated with a large parapapillary gamma zone, the MBMD was not found in vertically directed OCT scans, but in horizontally orientated or obliquely directed scans (Fig 3). In five eyes, MBMDs of different types were detected (Fig 4).

## Discussion

The results of our study on highly myopic individuals suggested that some DSMs were associated with MBMDs. In eyes with a DSM incompletely surrounded by a MBMD, as it was typically the case in eyes with DSM and a MBMD associated with patchy atrophy, the retina, RPE and choroid appeared relatively unchanged in the central region with BM preserved (Fig 1). In the ring-like BM-free region partially surrounding the central prominent island of the DSM in these eyes, the RPE, the outer and middle retinal layers, the choriocapillaris and the middle-sized choroidal vessel layer were absent. From an anatomical point of view, it suggested that visual acuity in the region of the DSM as a central island, in particular in eyes without additional CNV, was relatively preserved, while a deep scotoma might have been present in the ring-like periphery of the central island due to the loss of photoreceptors (Figs 1–3).[16] From an etiological point of view, the morphology of a DSM as a prominent central island incompletely surrounded by a ring-like MBMD may make one infer that a MBMD-associated change in the biomechanics in the posterior ocular wall, in addition to other factors such as size and location of chorioretinal atrophic areas at the posterior pole, biomechanical tissue parameters of the posterior sclera, intraocular asymmetries in axial elongation and orbital tissue pressure, may have played a role in the pathogenesis of some DSMs.

The finding that the retinal layers with an intact central BM within the area of the DSM appeared relatively unchanged on the OCT images was in agreement with recent studies in which the retinal thickness in the macula in eyes without myopic maculopathy was independent of axial length.[17] Correspondingly, the density of the RPE cells in the macular region was not related to axial length in a recent histomorphometric investigation.[18] However, the axial elongation was associated with an increase in the disc-fovea distance due to an enlargement of parapapillary, BM-free, gamma zone.[19] The results of the previous investigations together with the findings of our study suggest that the presence of an intact BM is protective against axial elongation associated changes in the overlying retina.

The findings of the present study on an association between DSMs and MBMDs may add another element to the discussion on the etiology of DSMs, which has remained elusive so far. Although the pathogenesis of MBMDs has been unclear, one may discuss that the myopic enlargement of the globe overstresses the stability of BM close to the posterior pole and leads to a ruptures of, and to the development of holes in, BM in the paracentral region. If these BM ruptures and MBMDs occur in a circular manner around the fovea, a central island of intact BM is formed surrounded by MBMDs in the pericentral region. In the case of an incomplete circular arrangement of the MBMD, a central peninsula of intact BM develops. Since BM may have some biomechanical properties in terms of strength in stress and strain,[20] the almost circular defect in BM may release the central island from biomechanical forces so that it can slightly bulge inward. If this inward protrusion of the central island is more than 50 μm measured from the base line of the RPE, it is called DSM. Besides this hypothetical, on MBMDs based mechanism, other etiologies may without doubt play a role in the development of DSMs. It includes the influence of a local thickening of the subfoveal sclera as described by Imamura, Spaide and coworkers.[3] It was discussed that by shortening the optical axis, the focally thickened subfoveal sclera was associated with a response to the myopic defocus of the image at the posterior pole in highly myopic eyes[4–6] Other potential mechanisms involved in the pathogenesis of DSM may include biomechanical sequels of chorioretinal atrophic areas at the posterior pole, changes in the biomechanical parameters of the posterior sclera including a regional stiffening or regional weakening, orbital tissue pressure, and forces exerted by an abnormal insertion of the oblique extraocular muscles close to the posterior pole.

Chorioretinal neovascularization can occur along the foveal edge of patchy atrophy in highly myopic eyes if the patchy atrophy is located close to the fovea.[21] Eyes with DSM and MBMDs in association with patchy atrophy may therefore have a risk of developing CNV along the foveal edge of MBMDs, what may be addressed in future studies.

Potential limitations of our study should be discussed. First, the study participants were recruited in a highly specialized third-referral clinic for high myopia so that it has remained unclear whether the findings obtained in our study are representative for DSM in general. Second, the hypothesis of BM as the primary expanding structure in the process of emmetropization and myopization is speculative, despite some histological and clinical findings supporting it. Third, about a half of the eyes with a DSM did not have detected MBMDs, so that the question arises why these eyes developed a DSM although BM appeared to be intact. One may infer that the development of MBMD may be one among several mechanisms leading to a DSM. Fourth, the relatively high prevalence of MBMDs in our total study population as compared to the population of previous investigations may have been due to the design of the OCT examination, since we routinely performed 12 radial scans centered on the macula while in previous studies often only one vertical scan and one horizontal scan were assessed.[1, 3, 14, 22, 23] Fifth, it has remained unclear why some MBMDs were associated with a CNV while the MBMDs in the area of a patchy chorioretinal atrophy did not develop a CNV although BM as structure separating the choroid from the subretinal space was missing in both situations. Patchy atrophy develops away from the fovea (generally superior or inferior upper to the fovea), and when it enlarges it usually does not involve the foveola.[16, 24] In this type, MBMDs were usually found upper to and lower to the fovea so that the foveal BM remained as central island. The type of MBMD located in the area of a CNV-related macular atrophy was different from patchy chorioretinal atrophy, since it started in the foveal region around the CNV and enlarged circumferentially with time passing by.[16, 25] The third type of MBMD occurred as part of a temporally enlarging parapapillary gamma zone extending into the perifoveal region. This type of MBMD was neither associated with the development of CNV. Sixth, since our study had a cross-sectional design, any associations found do not allow to be taken as an evidence for a causal relationship. In particular, it has thus thus remained unclear whether MBMDs preceded the formation of DSM.

In conclusion, presence of a DSM was significantly associated with the presence of MBMDs. Within the region of a DSM with the BM preserved, the retina, the RPE and the choroid were relatively unchanged if there was no additional CNV. In the ring-like BM-free region partially surrounding the central prominent island or peninsula of a DSM, the RPE, the outer and middle retinal layers, the choriocapillaris and the middle-sized vessel layer of the choroid were absent. The morphology of the DSM in association with MBMDs may make one infer that the MBMDs led to a focal relaxation of the posterior sclera allowing the sclera to partially bulge inward and leading to the formation of a DSM.
